# Viscoelastic Behaviour of Flexible Thermoplastic Polyurethane Additively Manufactured Parts: Influence of Inner-Structure Design Factors

**DOI:** 10.3390/polym13142365

**Published:** 2021-07-19

**Authors:** Fernández Pelayo, David Blanco, Pedro Fernández, Javier González, Natalia Beltrán

**Affiliations:** Department of Construction and Manufacturing Engineering, University of Oviedo, 33213 Gijón, Spain; fernandezpelayo@uniovi.es (F.P.); dbf@uniovi.es (D.B.); javiergonal@gmail.com (J.G.); nataliabeltran@uniovi.es (N.B.)

**Keywords:** additive manufacturing, thermoplastic polyurethane, inner structure, mechanical properties, viscoelasticity

## Abstract

Material extrusion based additive manufacturing is used to make three dimensional parts by means of layer-upon-layer deposition. There is a growing variety of polymers that can be processed with material extrusion. Thermoplastic polyurethanes allow manufacturing flexible parts that can be used in soft robotics, wearables and flexible electronics applications. Moreover, these flexible materials also present a certain degree of viscoelasticity. One of the main drawbacks of material extrusion is that decisions related to specific manufacturing configurations, such as the inner-structure design, shall affect the final mechanical behaviour of the flexible part. In this study, the influence of inner-structure design factors upon the viscoelastic relaxation modulus, *E*(*t*), of polyurethane parts is firstly analysed. The obtained results indicate that wall thickness has a higher influence upon *E*(*t*) than other inner-design factors. Moreover, an inadequate combination of those factors could reduce *E*(*t*) to a small fraction of that expected for an equivalent moulded part. Next, a viscoelastic material model is proposed and implemented using finite element modelling. This model is based on a generalized Maxwell model and contemplates the inner-structure design. The results show the viability of this approach to model the mechanical behaviour of parts manufactured with material extrusion additive manufacturing.

## 1. Introduction

Continuous advances in additive manufacturing are broadening the field of application of resultant parts from mere prototypes to industrial-quality products [1]. During the last decade, sustained growth of additive manufacturing has evolved from a desktop-3D-printing oriented market to an increasing adoption of industrial additive manufacturing systems [2]. Although power-bed fusion and directed energy deposition processes have experienced a great impulse, MEX (extrusion based additive manufacturing) is still the most popular and widely used category of additive processes. In MEX, material is “selectively dispensed through a nozzle” to make parts layer upon layer [3]. Processes in this category commonly use a thermoplastic material as the feedstock [1]. 

Mechanical properties and structural reliability of additively manufactured parts have been under study for the past two decades [4]. It is well known that the layer-upon-layer deposition strategy leads to anisotropic properties and that additively manufactured parts present lower tensile strengths than the equivalent parts fabricated by injection moulding, due to voids formation and internal structure characteristics [5]. 

Moreover, Forster [6] discuss the inherent complexity of relating material properties in additive manufacturing, since manufacturers tend to determine the properties of a particular design, used as an example, rather than to establish standardized methods. The author points out that even when this issue is critical, the literature available is not significantly large. Nevertheless, influence of inner-design manufacturing parameters (such as layer height or infill percentage) on the mechanical behaviour of rigid parts is not an unusual research concern [7].

Flexible filaments constitute a particular category of MEX materials, since their properties make them suitable for a wide range of applications (impact energy absorption [8] and integration of wireless components into flexible devices [9] or creation of biomechanical components [10]). The possibility of building multimaterial parts to create functionally graded materials is another promising capacity [11].

The influence of inner-design manufacturing parameters in single-material flexible parts has been partially addressed by Plott et al. [12]. This research evaluates the ultimate tensile strength of silicone dumbbell specimens fabricated by MEX. They found that tangency voids inside the material, especially those related to 0° infill orientation, worsen the mechanical performance of the specimens. Nevertheless, they also indicate that once the presence of elongated voids is minimized, orthogonal (90 °) or angular (± 45 °) infill orientations would provide similar results in terms of tensile strength, whereas parallel orientations clearly provided worse results.

In the field of flexible filaments, thermoplastic polyurethanes (TPU) occupy a niche between elastomers and other polymers since they can provide the mechanical performance of rubber while being thermoplastic processes [13]. Therefore, the use of single-material TPU parts is relevant for many applications and several research items can be found on this subject [14,15]. Influence of inner design manufacturing features has been considered in a recent work by Nakajima et al. [16], although in this case a “solid” (100% infill) arrangement was used.

Nevertheless, research regarding mechanical characterization of TPU parts is more intensive in the field of composites and multimaterial parts [17,18,19], where specific issues, like the influence of boundaries between materials [13], significantly affect the mechanical behaviour of the part.

In summary, although the possibility of using flexible TPU in MEX processes provides an interesting range of solutions, the lack of models that consider inner-design manufacturing factors makes it difficult to predict how manufactured parts would behave under mechanical solicitations. In order to fill the gap of knowledge in this particular subject, present work evaluates viscoelastic behaviour of MEX parts built up in TPU. Differences in the mechanical behaviour of TPU filament before and after the extrusion are firstly analysed. Then, the behaviour of three-dimensional test specimens with different inner-design manufacturing characteristics is studied. A mathematical equation that describes such behaviour within the limits of the experiment is then used to simulate with a finite element model (FEM) the mechanical behaviour of a series of verification parts. Finally, discrepancies between model predictions and measured values are presented and discussed.

## 2. Materials and Methods

The final mechanical properties of TPU parts are greatly influenced by their viscoelastic behaviour [18,19], which means that they behave partially like an elastic material and partially like a viscous one [20].

To characterize a linear viscoelastic material under conditions of constant strain *ε*_0_, stress relaxation tests can be performed in order to obtain the stress relaxation modulus *E*(*t*) [20] with the following expression:

(1) $$E\left(t\right)=\frac{\sigma \left(t\right)}{\varepsilon_{0}}$$

where *σ*(*t*) is the experimental stress obtained during testing. In this work, the relaxation modulus, *E*(*t*), was measured on a RSA3 Dynamic Mechanical Analyser (TA Instruments, Delaware, EEUU) under a controlled laboratory temperature (20 °C ± 0.5).

Commercial TPU brand Filaflex 82A, manufactured by Recreus Industries S-L (Alicante, Spain, EU) was used in this work. This flexible material is presented in 2.85 mm diameter filament coils and has been conceived for being used in MEX processes. Examples of its use in different fields can be found in several research works [10,19]. A list of FILAFLEX most important properties, according to the supplier, is provided on Table 1.

**Figure 1:**
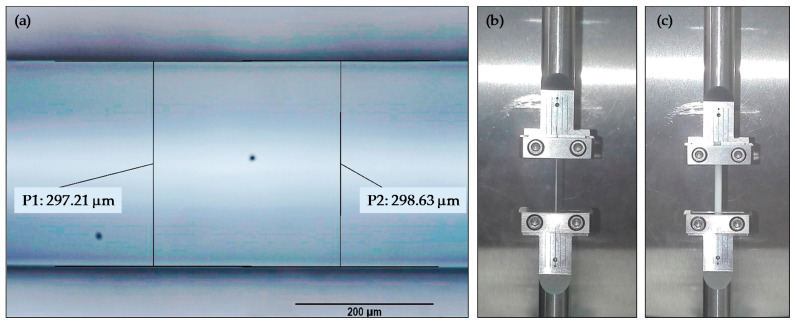
(**a**) Extruded TPU diameter measuring using the image analyser; (**b**) mechanical testing of an extruded filament sample; (**c**) mechanical testing of a feedstock sample.

Test specimens were manufactured in a SIGMA R17 machine (BCN3D, Barcelona, Spain, EU) with a 0.4 mm diameter nozzle. Extruding TPUs with consumer grade additive manufacturing machines is not an easy task, at least when compared with more rigid materials, like polylactic acid or acrylonitrile butadiene styrene. Among the usual recommendations for working with this type of materials is to employ moderate speeds and to select an antibuckling extrusion mechanism adapted for flexible materials. In this work, standardized manufacturing configuration provided by BCN3D (Table 2) was used in CURA (an open-source slicing software for additive manufacturing machines) to generate a manufacturing code. Slight differences in the diameter of the extruded material were noticed, probably caused by variations in extrusion speed related to process dynamics. An Image Analyser (NIKON, EPIPHOT 200, Minato, Japan) was used to calculate an average diameter of 0.298 mm for the extruded TPU (Figure 1a).

### 2.1. Tests Conducted on Feedstock and Extruded Material

Two different types of tests were conducted to evaluate differences in TPU behaviour between feedstock and extruded material. Firstly, three consecutive loading cycles were applied to the test filaments, so that the strain and stress curves were obtained. The tests were carried out in the RSA3 equipment. Each cycle consists of a quasistatic tension tests at 3 mm/min until maximum load capacity of the RSA3 equipment (35 N) and a downloading step until zero load at the same displacement ratio. A waiting time of 1 h was used before the next cycle to allow the material to recover. Secondly, relaxation tests were conducted in the RSA3 equipment. A strain level of 1% was used in the tests and five specimens were used for both feedstock and processed filaments. Figure 1c provides an image of feedstock testing whereas Figure 1b corresponds to an already extruded filament.

### 2.2. Test Conducted on Manufactured Specimens

**Figure 2:**
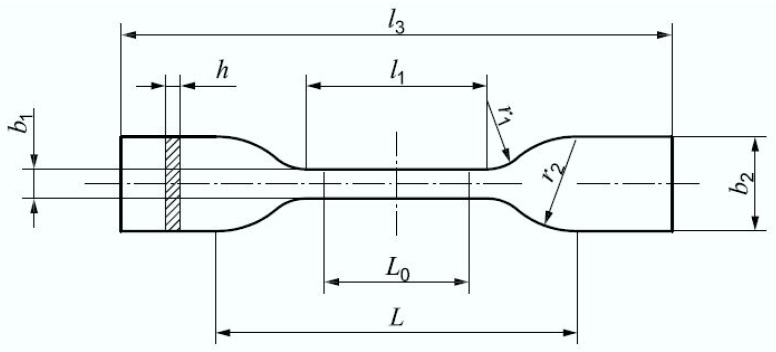
ISO 527-2 type 5A test specimen.

To evaluate the influence of inner-design factors, a part resembling the shape of a Type 5A test specimen was used [21]. Although it has not been specially designed for additive manufacturing processes, Forster [6] indicates that this standard could be applicable for additive manufacturing testing. This specimen follows the traditional dog-bone shape (Figure 2), while its dimensions can be found in Table 3.

**Figure 3:**
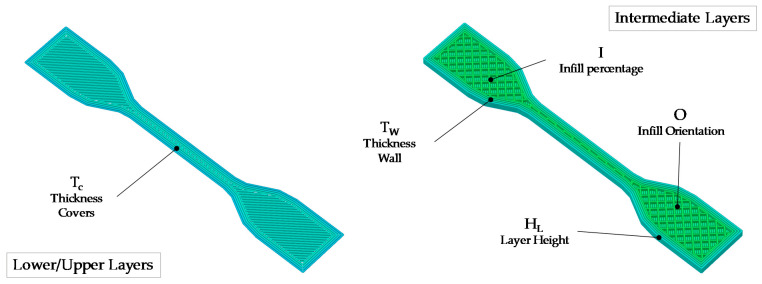
Inner-design factors in MEX (*T_C_*: thickness of covers, *T_w_*: thickness of the wall, *I*: infill percentage, *H_L_*: layer height, *O*: infill orientation).

The inner-design of the part is defined through the parameterization of two volumes: the shell (external volume) and the infill (internal volume). The shell forms a solid volume that occupies the periphery of the part and can be defined as a combination of solid layers (lower and upper sections of the part) with a solid volume comprised between the external contours of every intermediate layer and their correspondent internal offsets. Consequently, the thickness of covers (*T_C_*) determines the number of layers that form the upper and lower solid sections of the part, whereas the thickness of the wall (*T_W_*) fixes the offset between external and internal contours for intermediate sections. The structure of lightened areas inside the intermediate sections is defined by the morphology and structure of the infill. Firstly, the infill percentage (*I*) establishes a proportion between the volume of material and the void volume for lightened areas; secondly, the type of infill determines the pattern that is used to deposit material inside those areas. In this work, a “grid” pattern has been used, since it provides squared-lattice reinforcements (which is a better solution than linear patterns, especially for low infill values) while not compromising manufacturing times (associated with complex infill types like “honeycomb” or “triangular”). Nevertheless, this type of infill can be also modified with an infill orientation parameter (*O*) that fixes the angle between the orientation of the grid and the Cartesian reference system of the layer. Finally, layer height (*H_L_*) defines the vertical distance between layers, affecting both the shell and the infill. A visual representation of inner-design parameters for solid and lightened sections of the test specimen is provided in Figure 3.

The goal of this work was not just to determine the relative significance of these five parameters upon the viscoelastic behaviour of lightened specimens, but to provide a mathematical model capable of predicting how parts would behave as a function of inner-design factors. Accordingly, a two-level fractional factorial (2^5−1^) DOE was selected. This resolution V design requires only 16 experimental runs, whereas it guarantees no aliasing between the main effects and two-factor interactions. To estimate the experimental error, two replicates were considered, so that the final DOE consisted of 32 experimental runs. A 0.001 α-level was considered for the analysis of variance to minimize false positives (Type I errors), which is to say: wrongly identifying a factor or an interaction as significant. This experimental arrangement was designed and analysed using Minitab^®^ 17.1.0. Table 4 contains the low and high values of each DOE factor considered within the scope of this research.

**Figure 4:**
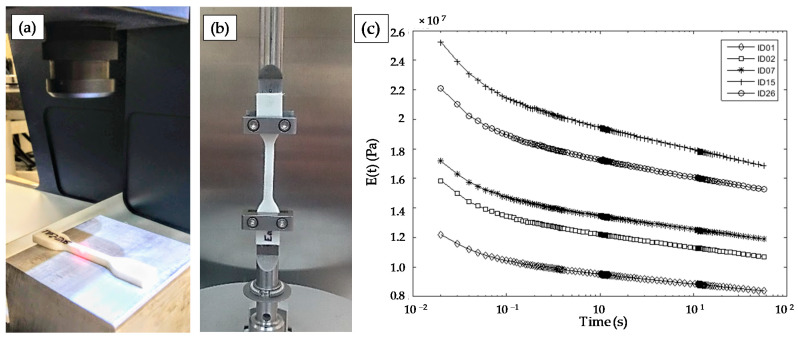
(**a**) Test specimen measuring using the conoscopic equipment; (**b**) mechanical testing of the specimen on the RSA3 equipment; (**c**) relaxation curves obtained during pretest work for different samples.

Once each test specimen was manufactured, its actual dimensions were measured using a conoscopic sensor (CONOSCAN 4000, Optimet, Jerusalem, Israel) with a 50 mm lens. This configuration provides an accuracy lower than 6 µm, according to the manufacturer (Figure 4a). This non-contact digitizing technique was selected to prevent introducing an additional uncertainty related to the deformation of test samples caused by contact methods. Finally, test samples were subjected to relaxation tests under tension (Figure 4b) at the reference temperature, using a 1% strain. Relaxation curves in Figure 4c, obtained for five different specimens used during pretest adjustments, present similar relaxation behaviour with the only difference of vertical shifts between them. These shifts reflect the influence of inner-design differences between tested specimens.

Since a numeric value was required for the response used in the DOE, it was decided that the value of *E*(*t*) when the relaxation test reaches ten seconds (*E*(*10*)) would be employed as an indicator of mechanical behaviour.

## 3. Results

### 3.1. Comparison between Feedstock and the Extruded Filament

**Figure 5:**
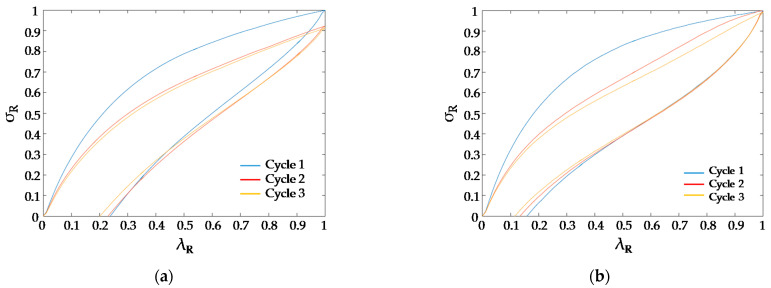
Stress–strain cycles. (**a**) For the feedstock filament; (**b**) for the extruded filament.

To compare their respective behaviour, feedstock and extruded filament specimens were subjected to 3 loading cycles. Each corresponding stress–strain cycle was normalized as follows: σ_R_ = σ/σ_1max_ and λ_R_ = ε/ε_1max_, where σ_1max_ and ε_1max_ are the maximum stress and strain values, respectively, for the first cycle. Results from the 3 loading cycles upon the feedstock filament is provided in Figure 5, where it can be inferred that a “softening stress” phenomenon was affecting the material from the first and subsequent cycles. The maximum stress decreased from cycle to cycle and there was a hysteresis loop in the material. Since this behaviour has already been observed for cast TPU [16], it can be assumed that it is reflecting an initial damage in the material induced by the Mullins effect [22] followed by the common elastomeric hysteresis loop [18].

The load cycles observed for the feedstock were quite different from those obtained for the extruded filament (Figure 5). Firstly, the loading and unloading curves were different; secondly, stress values reached a similar value for the extruded filament in all cycles. Additionally, there was a slight Mullins effect from the second to the third cycle in the extruded material that was negligible in the feedstock material. Finally, the extruded filament shows an improved recovery capacity (*λ*_R_ = 0.15) with respect to the feedstock (λ_R_ = 0.24). These facts imply that a change in the mechanical behaviour of the TPU occurred when the material was processed.

**Figure 6:**
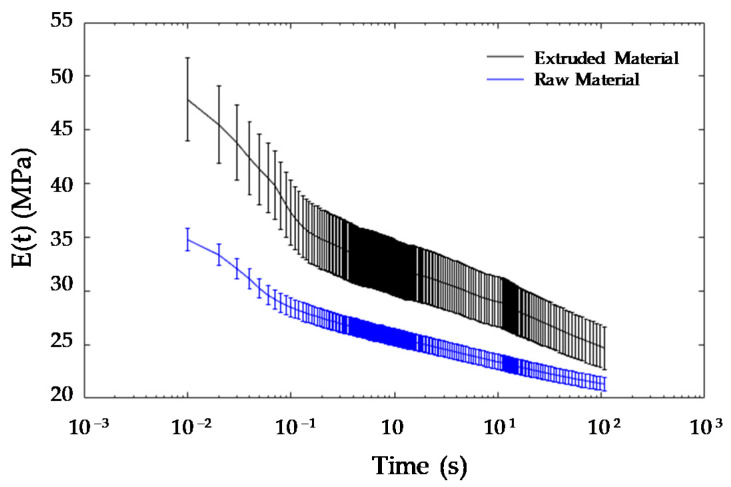
Relaxation curves obtained for the tested filaments.

Considering the previous results, four cycles at the 10% strain level were applied to the filament samples (both feedstock and extruded) as a preparation step before conducting the relaxation tests, in order to remove the possible influence of the Mullins effect in the results. The averaged relaxation curves for both filaments are presented in Figure 6.

Figure 6 shows how the extruded material presents a higher modulus than that obtained for the feedstock filament. It can also be noticed that the extruded material presents a higher scattering than the one observed in the feedstock. This effect could be related to the difficulty of obtaining a uniform diameter for all the length of the extruded filament, since the dynamics of the feeding mechanism does not provide a perfectly steady filament movement. Conversely, a higher percentage of relaxation for the total testing time (46%) can be also observed in the extruded material, when compared to that measured for the feedstock material (38%). The relaxation test also pointed to a different behaviour in the mechanical properties after the feedstock is extruded.

### 3.2. Analysis of Inner-Design Factors Influence

ISO 527-2 Type 5A test specimens were manufactured and E(t) curves obtained with the RSA3 equipment. Table 5 contains values, extracted from these experiments, for every single run within the DOE. Additionally, dimensions $b_{1}$ and h (Figure 2) were measured without contact with the conoscopic sensor for each manufactured test specimen and correspondent results are also provided in the same table.

The analysis of variance (Table 6) reflects that all individual factors within the DOE had a significant influence upon the response E(10). On the other hand, none of the two-way interactions between those factors present a significant influence. Additionally, the F-value provides a ratio amid the between-groups variance and the within-groups variance, so that a relatively high F-value means that the observed variability for that factor explains a large amount of the experimental variability. Accordingly, it can be asserted that the existence of a significant influence of each individual factor upon the variance of E(10) within the experimental range cannot be rejected.

**Figure 7:**
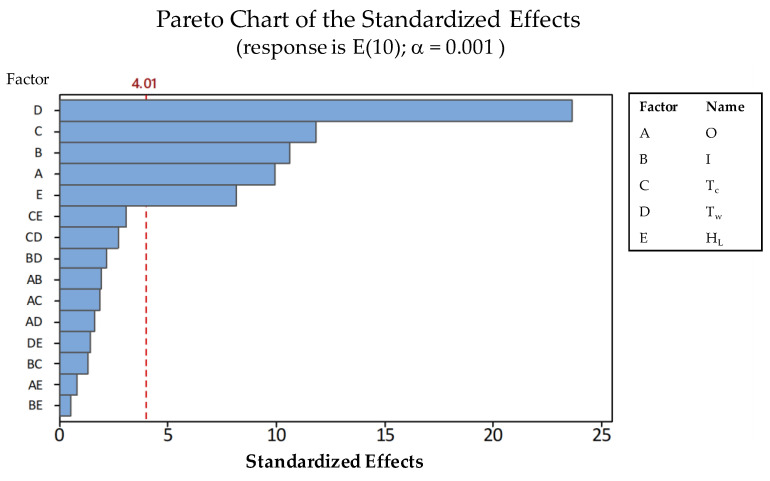
Pareto chart of the standardized effects.

The relative importance of individual influences can be better understood by means of the correspondent Pareto chart (Figure 7).

**Figure 8:**
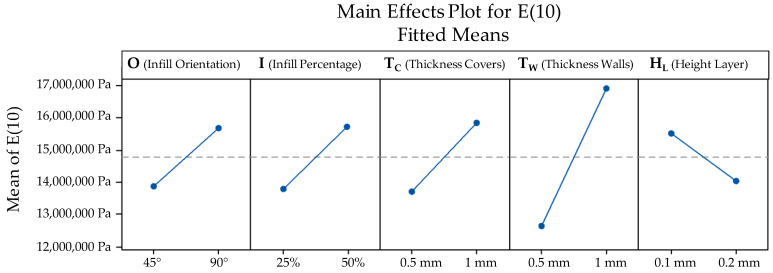
Main effects plot for E(10).

It can be asserted that $T_{W}$ had the biggest influence upon the variability of E(10), followed (in order of significance) by $T_{C}$, I, O and $H_{L}$. The relative effect of variations between low and high values for each parameter (according to Table 4) upon fitted means can be seen in Figure 8. The influence of $T_{W}$ stands out clearly, indicating that thickening the walls of the part shall significantly increase its mechanical resistance. $T_{W}$, $T_{C}$ and I all show a logical influence upon E(10), since an increase in their value causes an increase in the resistance. The orientation of the internal grid does also influence the results, so that grids parallel to the longitudinal axis (90°) are preferable than those oriented at 45°. On the other hand, lower values for the layer height are preferable since they produce slightly higher values for E(10).

Minitab provided the following regression equation, based on the experiments:

(2) $$\begin{array}{ll} E\left(10\right)=\; & -4,759,435+47,838\times O+98,541\times I+13,986,089\times T_{c}\\ & +14,082,830\times T_{W}-12,734,837\times H_{L}+620\times O\times I\\ & -29,325\times O\times T_{c}-25,476\;O\times T_{w}+66,268\times O\times H_{L}\\ & -37,243\times I\times T_{c}-62,511\times I\times T_{W}+74,876\times I\times H_{L}\\ & -3,927,196\times T_{c}\times T_{W}-22,460,747\times T_{c}\times H_{L}+10,019,217\\ & \times T_{W}\times H_{L} \end{array}$$

This model shows excellent adjustment, since the calculated value for S (standard deviation of data values from the regression line) was 51.255 kPa, whereas the coefficient of determination R-sq (proportion of variation in the response data that is explained by the predictors in the model) was 98.45%. The R-sq(adj) was 96.99 %, while the R-sq(pred) (a modified R-sq that reflects how well the model predicts future data) was 93.78%. This fitted model can be used to predict the viscoelastic behaviour of parts with values of internal design factors different from those already tested, within the limits of the experimental range.

## 4. Modelling and Validation

**Figure 9:**
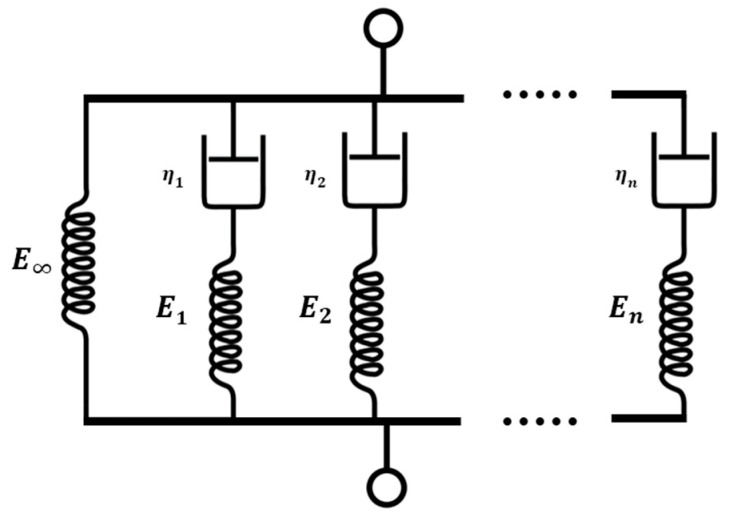
Generalized Maxwell model.

To illustrate a practical application of the fitted model obtained from DOE, a FEM was assembled in ABAQUS. The great advantage of the fitted model is that lightened parts can be assimilated to material-equivalent solid parts since the influence of the design parameters was included in the material model according to Equation (2). The first step was to fit the viscoelastic relaxation behaviour of the material. As was mentioned previously, the curves for the different design parameters were almost the same but shifted in vertical. Therefore, one of the curves, in this case, the *ID06* relaxation curve, was fitted using a generalized Maxwell model (Figure 9).

The coefficients of the model were fitted using Prony series [23] by means of the following equation:

(3) $$E\left(t\right)={\;E}_{0}\left[1-\overset{n_{t}}{\underset{i\;=1}{\sum }}e_{i}\left(1-\exp\left(-\frac{t}{{\;\tau }_{i}}\right)\right)\right]$$

where $E_{0}$ is the instantaneous modulus, and $e_{i}$ and $\tau_{i}$ are the Prony coefficients. Using the fitting toolbox of MATLAB, the relaxation modulus was fitted with eight terms being the correlation factor $R^{2}=0.9982$. The values of the Prony coefficients are present in Table 7.

**Figure 10:**
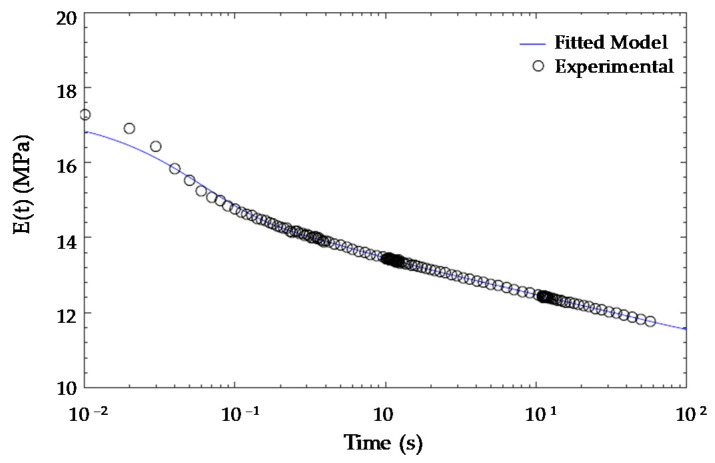
Generalized Maxwell model fitted and experimental relaxation modulus for the ID06 specimen.

Finally, the fitted viscoelastic model particularized for ID06 is presented in Figure 10.

As it can be seen, the relaxation modulus fitted with Equation (3), depends on the instantaneous modulus, which is the Young’s modulus of the material at time t=0 s. On the other hand, the Young’s modulus for t=10 s was used in the fitted model for the lightened specimens (Equation (2)). For this material, the average ratio between both moduli for the 32 specimens tested was:

(4) $$\frac{E_{t=0s}}{E_{t=10s}}=1.390\pm 0.018$$

which corresponds with an approximate relaxation rate of 72 % after 10 s.

Once the model was completed, a validation test was performed to check its capability to predict the behaviour of newly defined parts (not previously tested), whose design parameters fall into the experimental range. Therefore, a new set of specimens were designed and manufactured. It has been decided that three combinations of values, providing comparatively high, low and average values of E(t) shall be tested. For each combination, two identical specimens were manufactured and tested. Table 8 contains the values adopted for each combination of factors (V1, V2 and V3) in the validation specimens. Since each specimen was manufactured twice, the set of specimens finally consisted of six parts (V11, V12; V21, V22; V31 and V32).

**Figure 11:**
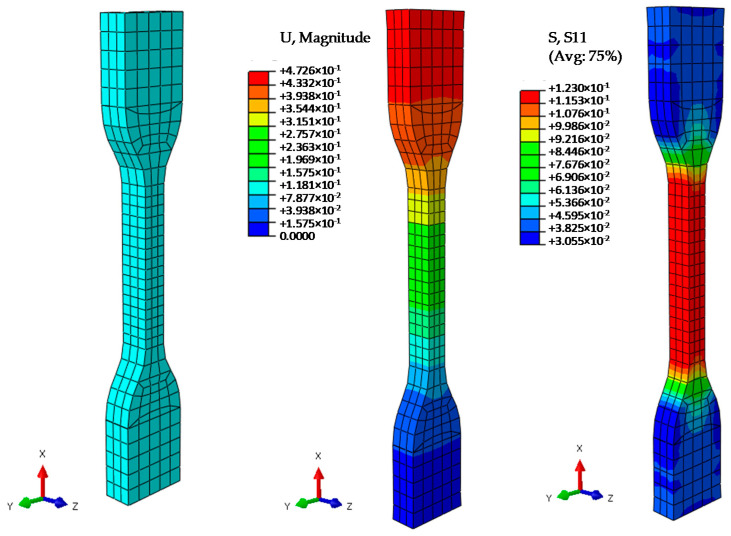
Finite element model and examples of the results obtained.

The finite element model and an example of the results obtained are presented in Figure 11.

**Figure 12:**
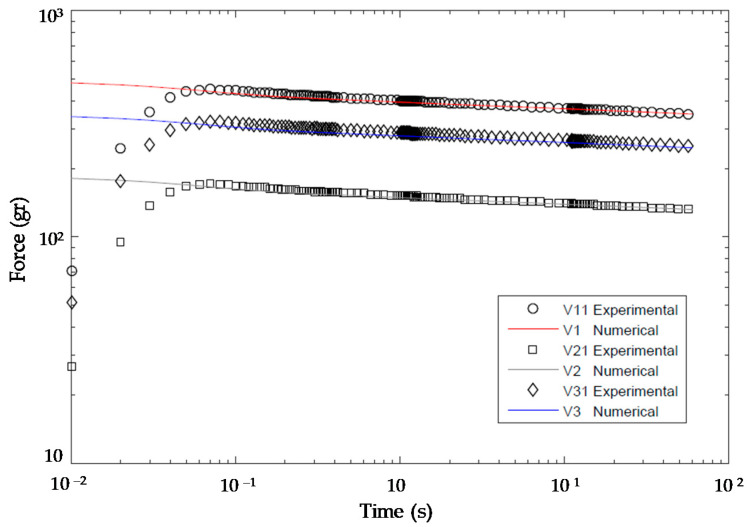
Comparison between the experimental forces and those obtained from viscoelastic FEA.

The complete curves for each simulation V1, V2 and V3 are presented alongside with experimental results V11, V21 and V31 in Figure 12.

As expected, the average errors obtained are of the same order of those obtained in the validation process for time t=10 s, being approximately 2%, 5% and 1% for V11, V21 and 31, respectively. In Figure 12, it can also be seen that a noticeable difference exists between the experimental force and the simulated one for the three first values in all curves. These differences are related to the impossibility of applying an instantaneous strain step in the RSA3 testing machine, whereas simulating this ideal condition in the finite element model is possible. Table 9 contains the distribution of measured values of the quality indicator ($E{\left(10\right)}_{M}$) and their correspondent ones predicted with the model in Equation (5), ($E{\left(10\right)}_{P}$), alongside with the error observed in each case.

Average error within the validation set was 0.148 MPa, which represents a 0.218 % average deviation for the model prediction with respect to the measured values. Therefore, it can be asserted that the model is capable of accurately predicting the value of E(10) for specimens within the experimental range.

## 5. Discussion

Firstly, both feedstock and the extruded filament showed properties common to elastomers. An initial damage in the materials was present under the loading cycles, overall, from the first to second loading cycles. This behaviour can be related to the Mullins effect [22], which has been also reported by Patton et al. [18]. Differences in the stress–strain curves were also found in the extruded filament in relation to the feedstock. This result implies that the mechanical properties were modified during the extrusion of the feedstock material. Although it is known that extrusion parameters have an effect on the mechanical properties of extruded materials [7,23], further investigations must be carried out in extruded TPUs. As regards the relaxation modulus obtained in the extruded filament, the values ranged from 26 to 48 MPa. Although these values cannot be directly compared [20], the range covered the storage modulus value (38 MPa) reported by the material manufacturer.

Secondly, handling of specimens during dimensional measurement procedures showed that contact measuring methods were not appropriate for additively manufactured TPU flexible parts. It was clear during the preparatory work that using mechanical micrometres for the contact measurement of part dimensions could induce a degree of deflexion that would affect the quality of results. In fact, this effect was extremely large when measuring parts with high degrees of lightening. Since the actual (measured) dimensions were used in the mechanical parameters calculation, this circumstance was addressed by using a non-contact measurement (in this case, a conoscopic holography device). Accordingly, this circumstance should be taken into account in future research in the field of flexible MEX, in order to avoid using noisy values for model construction.

Thirdly, it was observed that an adequate selection of the inner-design parameter had a positive effect upon mechanical properties, e.g., increasing the infill to obtain higher stiffness could be an inadequate strategy, when compared to increasing the thickness of the wall. Designers should analyse the balance between manufacturing time/cost or part weight and the effective improvement on part stiffness. Considerations like this must be incorporated to the overall design process, so that designers become aware of the relevance of inner design upon the part´s final performance. Optimization of part design should not only be carried out based on the external shape. Additionally, in the case of MEX parts, inner design factors should always become part of the overall optimization process.

Finally, a viscoelastic model that covers the range of the studied parameters was presented. The viscoelastic TPUs material model allows for the prediction of the mechanical behaviour of the manufactured TPUs parts with good accuracy. As the TPU viscoelastic model considers the studied parameters, the manufactured parts can be modelled successfully with the solid homogenous section in FEM simulations. Although further studies must be carried out in FEM modelling of MEX, material models that cover the influence of the parameters of manufacturing can be very useful due to the advantages and simplicity of using a solid homogeneous section against modelling the exact geometry of the part.

## 6. Conclusions

The influence of inner-structure design factors upon the final mechanical properties of MEX TPU parts cannot be neglected and, therefore, they should be carefully considered during the selection of an optimal manufacturing configuration.

TPU materials present a viscoelastic behaviour. Moreover, significance differences between feedstock and extruded material behaviour were observed. Extruded material presents a higher modulus than feedstock, but also a higher relaxation ratio.

The design of experiments (DOE) revealed that the wall thickness ($T_{W}$) had the largest influence between the different inner-structure design parameters analysed in this work. Therefore, a smart design of inner structure could improve mechanical properties of TPU parts without negatively affecting manufacturing time or part weight.

A relationship between the inner-design parameters and the Young’s modulus *E(t)* of the specimens at a certain time was established by means of an equation. This equation was later extended by means of a generalized viscoelastic Maxwell model to expand the model in the time domain.

Finally, a finite element model was used to predict the behaviour of $E_{t}$ for a set of validation specimens. Prediction was founded to be in good agreement with experimental results, supporting the usefulness of this method to anticipate part behaviour and include inner-design factors as a regular specification for designers.

## Figures and Tables

**Table 1:** Standardized properties for Recreus Filaflex.

Property	Standard	Value	Unit	Test Condition
Shore hardness, Method A	ISO 868	82	--	
Ultimate Tensile Strength	DIN 53504	54	MPa	
Elongation to Break	DIN 53504	700	%	200 mm/min
Compression set	ISO 815	25	%	72 h; 23 °C
Impact resilience	ISO 4662	42	%	
Tensile storage modulus	ISO 6721-1,-4	48	MPa	20 °C
Tensile storage modulus	ISO 6721-1,-5	33	MPa	60 °C
Density	ISO 1183-1	1200	Kg/m^3^	
Extrusion-Melt Temperature		200 – 260	°C	

**Table 2:** Printing parameters for Recreus Filaflex.

Property	Value
Printing Temperatures	215 °C
Bed Temperature	65 °C
Printing Speed	20 mm/s
Extrusion Flow Rate	120 %
Retraction Distance	2.5 mm
Infill Overlap	10 %

**Table 3:** Test specimen dimensions according to ISO 527-2 type 5A.

Variable	Description	Dimension (mm)
$l_{3}$	Total length	75
$b_{2}\;$	Width at ends	12.5
$l_{1}$	Length of narrow parallel-sided portion	25
$b_{1}$	Width of narrow parallel-sided portion	4
$r_{1}$	Small radius	8
$r_{2}$	Large radius	12.5
h	Thickness	5

**Table 4:** Uncoded low and high values for each DOE factor.

	$T_{c}\;(\mathrm{mm})$	$T_{w}\;(\mathrm{mm})$	I	O	$H_{L}\;(\mathrm{mm})$
Low	0.5	0.5	25	45	0.1
High	1	1	50	90	0.2

**Table 5:** E(10) and dimensional measures for test specimens in DOE.

ID	O	I	$T_{C}$	$T_{W}$	$H_{L}$	$b_{1}(\mathrm{mm})$	h	E
1	45	25	0.5	0.5	0.2	3.73	4.84	8.843
2	90	25	0.5	0.5	0.1	3.86	4.99	11.304
3	45	50	0.5	0.5	0.1	3.9	5	11.443
4	90	50	0.5	0.5	0.2	3.89	4.95	13.459
5	45	25	1	0.5	0.1	3.93	4.9	13.620
6	90	25	1	0.5	0.2	3.91	4.87	12.459
7	45	50	1	0.5	0.2	3.85	4.89	12.548
8	90	50	1	0.5	0.1	3.92	4.96	17.255
9	45	25	0.5	1	0.1	3.86	4.98	14.418
10	90	25	0.5	1	0.2	3.91	4.92	15.260
11	45	50	0.5	1	0.2	3.94	5.04	15.429
12	90	50	0.5	1	0.1	3.99	5	17.576
13	45	25	1	1	0.2	3.89	4.96	15.295
14	90	25	1	1	0.1	4.02	4.99	18.258
15	45	50	1	1	0.1	3.96	4.95	17.941
16	90	50	1	1	0.2	4	5.02	18.405
17	45	25	0.5	0.5	0.2	3.44	4.35	7.770
18	90	25	0.5	0.5	0.1	3.87	4.9	11.960
19	45	50	0.5	0.5	0.1	3.86	4.99	12.193
20	90	50	0.5	0.5	0.2	3.89	4.93	13.555
21	45	25	1	0.5	0.1	3.96	5.03	13.339
22	90	25	1	0.5	0.2	3.88	4.9	12.542
23	45	50	1	0.5	0.2	3.89	4.97	12.991
24	90	50	1	0.5	0.1	3.95	4.93	16.884
25	45	25	0.5	1	0.1	3.82	4.99	15.427
26	90	25	0.5	1	0.2	3.82	4.9	16.048
27	45	50	0.5	1	0.2	3.91	4.97	15.612
28	90	50	0.5	1	0.1	3.99	5.07	18.990
29	45	25	1	1	0.2	3.91	4.97	16.048
30	90	25	1	1	0.1	4.01	5.08	18.514
31	45	50	1	1	0.1	3.98	4.99	19.176
32	90	50	1	1	0.2	3.98	4.97	18.378

**Table 6:** ANOVA of the experimental set.

Source			DF	Adj SS	Adj MS	F-Value	*p*-Value
Model			15	2.66 × 10^14^	1.77 × 10^13^	67.55	0.000
	Linear		5	2.57 × 10^14^	5.14 × 10^13^	195.54	0.000
		O	1	2.58 × 10^13^	2.58 × 10^13^	98.35	0.000
		I	1	2.95 × 10^13^	2.95 × 10^13^	112.33	0.000
		$T_{c}$	1	3.69 × 10^13^	3.69 × 10^13^	140.47	0.000
		$T_{w}$	1	1.47 × 10^14^	1.47 × 10^14^	559.98	0.000
		$H_{L}$	1	1.75 × 10^13^	1.75 × 10^13^	66.57	0.000
	2-Way Interactions		10	9.36 × 10^12^	9.36 × 10^11^	3.56	0.012
		O×I	1	9.74 × 10^11^	9.74 × 10^11^	3.71	0.072
		$O\times T_{c}$	1	8.71 × 10^11^	8.71 × 10^11^	3.31	0.087
		$O\times T_{w}$	1	6.57 × 10^11^	6.57 × 10^11^	2.5	0.133
		$O\times H_{L}$	1	1.78 × 10^11^	1.78 × 10^11^	0.68	0.423
		$I\times T_{c}$	1	4.33 × 10^11^	4.33 × 10^11^	1.65	0.217
		$I\times T_{w}$	1	1.22 × 10^11^	1.22 × 10^12^	4.65	0.047
		$I\times H_{L}$	1	7.01 × 10^11^	7.01 × 10^10^	0.27	0.613
		$T_{c}\times T_{w}$	1	1.93 × 10^12^	1.93 × 10^12^	7.34	0.015
		$T_{c}\times H_{L}$	1	2.52 × 10^12^	2.52 × 10^12^	9.6	0.007
		$T_{w}\times H_{L}$	1	5.02 × 10^11^	5.02 × 10^11^	1.91	0.186
Error			16	4.20 × 10^12^	2.63 × 10^11^		
Total			31	2.70 × 10^14^			

**Table 7:** Prony series coefficients.

$\tau_{i}\;\left(s\right)$	$e_{i}$
5.4063 × 10^−2^	0.14292
0.14614	2.1163 × 10^−2^
0.39504	2.9420 × 10^−2^
1.0678	3.5212 × 10^−2^
2.8866	1.3225 × 10^−2^
7.8028	3.6500 × 10^−2^
21.092	8.0567 × 10^−4^
57.015	6.3713 × 10^−2^

**Table 8:** Combination of factors used in the validation set.

ID-Validation	$O\;({}^{{}^{\circ}})$	I (%)	$T_{c}$ (mm)	$T_{w}$ (mm)	$H_{L}\;(\mathrm{mm})$
V1	90	50	1	1	0.1
V2	45	25	0.5	0.5	0.2
V3	68	37.5	0.75	0.75	0.15

**Table 9:** Comparison between $E{\left(10\right)}_{M}$ and ($E{\left(10\right)}_{P}$ for specimens in the validation set.

ID-Validation	$E{\left(10\right)}_{M}\;(\mathrm{MPa})$	$E{\left(10\right)}_{P}\;(\mathrm{MPa})$	Error (MPa)	Error (%)
V11	19.717	19.938	0.221	1.110
V12	18.785	19.938	1.153	5.782
V21	8.399	8.307	−0.092	−1.107
V22	8.650	8.307	−0.343	−4.129
V31	14.796	14.799	0.003	0.021
V32	14.854	14.799	−0.054	−0.367

## Data Availability

The study did not report any data.
